# Distribution of lipid transfer protein 1 (LTP1) epitopes associated with morphogenic events during somatic embryogenesis of *Arabidopsis thaliana*

**DOI:** 10.1007/s00299-012-1314-0

**Published:** 2012-07-21

**Authors:** Izabela Potocka, Timothy C. Baldwin, Ewa U. Kurczynska

**Affiliations:** 1 Laboratory of Cell Biology, University of Silesia, Jagiellońska 28, 40-032 Katowice, Poland; 2 School of Applied Sciences, University of Wolverhampton, Wulfruna Street, Wolverhampton, WV1 1SB UK

**Keywords:** *Arabidopsis thaliana*, Cell wall, Cuticle, Lipid transfer proteins, Somatic embryogenesis

## Abstract

Using immunocytochemical methods, at both the light and electron microscopic level, we have investigated the spatial and temporal distribution of lipid transfer protein 1 (LTP1) epitopes during the induction of somatic embryogenesis in explants of *Arabidopsis thaliana*. Immunofluorescence labelling demonstrated the presence of high levels of LTP1 epitopes within the proximal regions of the cotyledons (embryogenic regions) associated with particular morphogenetic events, including intense cell division activity, cotyledon swelling, cell loosening and callus formation. Precise analysis of the signal localization in protodermal and subprotodermal cells indicated that cells exhibiting features typical of embryogenic cells were strongly labelled, both in walls and the cytoplasm, while in the majority of meristematic-like cells no signal was observed. Staining with lipophilic dyes revealed a correlation between the distribution of LTP1 epitopes and lipid substances within the cell wall. Differences in label abundance and distribution between embryogenic and non-embryogenic regions of explants were studied in detail with the use of immunogold electron microscopy. The labelling was strongest in both the outer periclinal and anticlinal walls of the adaxial, protodermal cells of the proximal region of the cotyledon. The putative role(s) of lipid transfer proteins in the formation of lipid lamellae and in cell differentiation are discussed.

*Key message* Occurrence of lipid transfer protein 1 epitopes in *Arabidopsis* explant cells accompanies changes in cell fate and may be correlated with the deposition of lipid substances in the cell walls.

## Introduction

Somatic embryogenesis (SE) provides a model system for the investigation of cellular totipotency and for the observation of early differentiation events during plant embryo development (Zimmerman 1993; Kurczyńska et al. 2007). In contrast to zygotic embryogenesis, somatic embryogenesis can easily be observed; the external conditions of embryo development can be controlled and large quantities of embryos obtained (Quiroz-Figueroa et al. 2006). Knowledge of the underlying mechanisms controlling the developmental switch from the somatic to the embryogenic cell pathway, de facto meaning the change of cell fate, has increased dramatically in recent years, but we are still far from a complete understanding of this process.

The primary plant cell wall is a highly dynamic structure that constantly undergoes remodelling during cell growth, division and differentiation. The remodelling, which is expressed through modification of cell wall structure and composition, usually accompanies the morphogenetic processes leading to the development of organs and embryos (Fortes et al. 2002; Pedroso and Pais 1995). It has been shown previously that during organogenic nodule formation from internodes of *Humulus lupulus*, the pre-nodular cell walls accumulate both callose and cutin (Fortes et al. 2002). As the authors discuss, this facilitates the creation of a specific cellular environment, in which permeability of the cell walls is changed, which then leads to the cells reentering the cell cycle and to organ formation. It is also thought that similar modifications of cell wall composition give rise to competent cells during the very early stages of embryogenic cell differentiation (Dubois et al. 1990; Pedroso and Pais 1995; Verdeil et al. 2001). For example, during somatic embryogenesis of *Camellia japonica*, deposition of lipid substances takes place, which enables cells to separate from their surroundings (Pedroso and Pais 1995). Such physical and physiological isolation is a prerequisite for subsequent metabolic changes which, in turn, are followed by changes in cell differentiation.

It has been postulated (Hendriks et al. 1994) that lipid transfer proteins (LTPs) are involved in the process of cutin transport and polymerization. Plant LTPs were first described more than 30 years ago (Kader 1975) as being able to transfer phospholipids between cell endomembranes and to bind fatty acids in vitro. Further studies proved an extracellular localization for the LTPs (Thoma et al. 1993) and the presence of a signal peptide at their amino terminal end (Bernhard et al. 1991). To date, most important functions identified for LTPs are a role in defense against bacterial and fungal pathogens through participation in signalling pathways (Maldonado et al. 2002) or through direct inhibition of growth of these organisms (Molina et al. 1993), in adaptation to stressful environmental conditions (Cameron et al. 2006), in pollen tube adhesion to the transmitting tract of the style (Park et al. 2000), in loosening of cell wall during cell growth (Nieuwland et al. 2005), and in somatic embryogenesis (Sterk et al. 1991).

Expression of lipid transfer proteins is a well-known early marker for the induction of somatic embryogenesis in several model species including *Cichorium* hybrid ‘474’, *Medicago sativa*, *Picea abies* and *Daucus carota* (Blanckaert et al. 2002; Poulsen et al. 1996; Sabala et al. 2000; Sterk et al. 1991). The use of semi-automated video cell tracking has established that in carrot suspension cultures all somatic embryos are derived from *AtLTP1* luciferase expressing cell clusters, and that the pattern of *AtLTP1* luciferase expression is identical to the expression pattern of the endogenous carrot lipid transfer protein gene *EP2* (Toonen et al. 1997). As shown by Sterk et al. (1991) expression of LTP genes is tightly associated with the first differentiated tissue of somatic embryos, i.e. protoderm. This outer layer exerts a regulatory role in controlling cell expansion during embryo development and is essential for the continuation of the developmental process (Dodeman et al. 1997).

The objective of the current study was to analyse the spatial and temporal distribution of lipid transfer protein epitopes, recognized by an anti-AtLTP1 antibody in explants of *Arabidopsis thaliana* subjected to somatic embryogenesis induction. We focused on early cellular events occurring in the regions of explants which are thought to participate in the development of somatic embryos. Our aim was to answer to two questions: (1) whether LTPs are involved in the morphogenic processes which take place during SE, (2) whether there is a correlation between the presence of cell wall-associated LTP epitopes, the deposition of lipid substances within the cell wall and changes in cell fate. We also made an attempt to compare the occurrence and localization of LTP1 labelling in the cells of explants revealing either embryogenic or meristematic character, in order to additionally verify the potential role of LTPs in somatic embryogenesis.

## Materials and methods

### Plant material and culture conditions

*Arabidopsis thaliana* plants ecotype Col-0 (Nottingham Arabidopsis Stock Centre) were grown in a mixture of soil and vermiculite (1:1, v/v), in a growth chamber at 20–23 °C, with a 16-h light/8-h dark cycle. The somatic embryo induction was performed using a method adopted after Gaj (2001), i.e. immature zygotic embryos at the bent-cotyledon stage of development were excised from siliques and grown on Phytagel-solidified B5 medium (Gamborg et al. 1968), supplemented with 5 μM 2,4-dichlorophenoxyacetic acid, 20 g/l sucrose, and adjusted to pH 5.8. The cultures were then incubated at 23 °C under a 16-h photoperiod for 3 weeks.

### Tissue preparation

For histochemical and immunocytochemical procedures, ten or more explants were sampled daily during the culture period and fixed in a mixture of 4 % formaldehyde, 1 % glutaraldehyde, 0.1 % Triton X-100, 2 mM CaCl_2_ and 1 % sucrose in phosphate-buffered saline (PBS) at pH 7.2 for 24 h at 4 °C (Chen et al. 2006). After washing in PBS, the explants were dehydrated in a series of increasing concentrations of ethanol. The samples were then infiltrated and embedded in L.R. White resin (Polysciences) which was polymerized for 8 h at 50 °C. Longitudinal sections were obtained with a Leica EM UC6 ultramicrotome. For light microscopy, 0.5–1 µm sections were collected onto poly-l-lysine coated microscope slides (Menzel-Glaser). For transmission electron microscopy (TEM), ultrathin sections (90 nm) were collected onto formvar-coated nickel grids (300 mesh, Sigma). Some samples were also embedded in low-melting polyester wax (Steedman’s wax) as described by Vitha et al. (2000). Wax sections were cut to a thickness of 6 μm with an electronic rotary microtome HYRAX M 40 (Carl Zeiss MicroImaging GmbH) and collected onto poly-l-lysine coated microscope slides.

### Histochemical staining

For general histology, semi-thin L.R. White sections were stained with 0.1 % Toluidine Blue O (Sigma) in PBS and viewed by brightfield microscopy. The presence of lipid substances was detected in Steedman’s wax-embedded sections using Sudan Black B (Sigma-Aldrich) and Nile Red (Fluka). For Sudan Black B staining, dewaxed sections were rinsed with 50 % ethanol, immersed in a 1 % solution of Sudan Black B in 70 % ethanol for 20 min, washed with 50 % ethanol and distilled water and observed under brightfield illumination. For Nile Red staining, the sections were incubated for 10 min in a solution consisting of Nile Red stock (1 mg/ml in acetone) diluted 1:200 in distilled water, briefly rinsed with distilled water and observed by epifluorescence microscopy (excitation filter BP470-490, dichromatic mirror DM500, barrier filter BA520IF). For nuclei visualization, slides were mounted in Vectashield medium containing DAPI (Vector Laboratories) and viewed by epifluorescence microscopy (excitation filter BP360-370, dichromatic mirror DM400, barrier filter BA420). Microscopic observations were performed using an Olympus BX41 microscope equipped with an Olympus XC50 camera.

### Immunolabelling for light and transmission electron microscopy

The immunolabelling procedures were performed according to Chen et al. (2006). For light microscopy immunolabelling, the sections of L.R. White-embedded material were incubated in 1 % NaBH_4_ for 15 min and then washed thoroughly with PBS buffer (5 × 10 min). Non-specific binding sites were blocked with 2 % foetal calf serum (PAA Laboratories), 2 % bovine serum albumin (Sigma-Aldrich) and 0.1 % Triton X-100 in PBS for 30 min (blocking buffer). After blocking, the samples were incubated with the rabbit polyclonal anti-AtLTP1 antibody (purchased from Rose Biotechnology, Winchendon, MA, USA) diluted 1:200 in the above solution at 4 °C overnight. Sections were then washed with the blocking buffer (5 × 10 min) and incubated with the secondary antibody a Cy3-conjugated AffiniPure goat anti-rabbit IgG H + L (Jackson ImmunoResearch Laboratories) at 1:100 dilution in blocking buffer for 1 h at room temperature. Sections were washed with blocking buffer (5 × 10 min) and rinsed in PBS followed by sterile distilled water. As a negative control, the primary antibody was omitted and blocking buffer was applied, with all the other steps of the procedure included. A similar procedure was applied to Steedman’s wax sections. However, in this case NaBH_4_ was not used and in blocking buffer there were 2 % foetal calf serum and 2 % bovine serum albumin. Finally, the samples were mounted in Fluoro-Gel (Electron Microscopy Sciences, L.R. White sections) and Citifluor (Agar Scientific, Steedman’s wax sections) and observed under an Olympus BX41 epifluorescence microscope (excitation filter BP530-550, dichromatic mirror DM570, barrier filter BA590). Images were captured with an Olympus XC50 camera. In order to answer the question as to whether or not there is any correlation between the distribution of LTP1 epitopes and deposition of lipid substances in the cell wall, two consecutive sections of Steedman’s wax-embedded material were stained with the anti-AtLTP1 antibody (for LTP1 epitopes detection) and with Sudan Black B and/or Nile Red (for lipid substances detection), according to procedures described above.

For immunolabelling at the transmission electron microscope level, ultrathin sections were blocked in blocking buffer (10 % bovine serum albumin, 0.1 % Triton X-100 in PBS) for 30 min and treated with primary antibody diluted 1:20 in blocking buffer, overnight at 4 °C. The negative control was performed by omitting the primary antibody and incubation in buffer. Sections were washed in blocking buffer (5 × 5 min) and incubated in goat anti-rabbit secondary antibody conjugated to 12 nm gold (Jackson ImmunoResearch Laboratories) at room temperature for 1 h. Grids were washed in sterile distilled water and stained in 2 % aqueous uranyl acetate for 45 min, followed by Reynolds lead citrate for 7 min. Sections were viewed with a JEOL 1200EX transmission electron microscope (80 kV accelerating voltage).

At the light microscope level, serial sections through explants were examined for each day of the first week of culture and for days 9, 11, 13, 15 and 21. Eight to ten samples were examined for each developmental stage, except for day 13, for which three samples were taken into consideration. Representative examples are presented including both fluorescence and phase contrast microscopy to indicate the position of cell and/or organ borders. Selected samples were further studied at the transmission electron microscope level. The plates were constructed using Corel PHOTO-PAINT X3 and CorelDRAW X3.

### The signal localization in embryogenic and meristematic cells

A Chi-square statistical test of independence was applied to analyse frequencies of the signal presence in specific localizations of various types of cells. Three types of cells were taken into consideration, namely cells that exhibited features typical of embryogenic cells, localized in the adaxial protoderm and subprotoderm of the proximal part of the cotyledon (EC, *n* = 23), cells that exhibited features typical of meristematic cells, localized in the adaxial protoderm and subprotoderm of the proximal part of the cotyledon (MC, *n* = 24) and meristematic cells of the shoot apical meristem (M, *n* = 22). Meristematic and embryogenic cells were distinguished according to the cytological characteristics proposed by Verdeil et al. (2007). Specific categories for the signal presence were the following: cell wall (CW), cell wall and cytoplasm (CWC), cytoplasm (C), no labelling (NL). Cells with a clear signal observed in the mentioned compartments were chosen for the analysis.

### Quantification of immunogold label

Gold particles were quantified by hand in protodermal walls of both embryogenic (E) and nonembryogenic (NE) regions of the explants on randomly chosen electron micrographs (His et al. 2001). The area of cell wall was determined by the use of AnalySIS 3.0 software (Soft Imaging System Gmbh, Münster, Germany). For each region, the density of labelling was calculated from 10 micrographs and expressed as the mean number of gold particles per μm^2^. The Shapiro–Wilk goodness-of-fit statistical test was applied to check normality of distribution of the labelling density in both regions. Both samples demonstrated normal distribution. So, the Student’s *t* test was used to check for statistically significant differences in labelling intensity between the two regions. The statistical analysis was performed using Statistica version 9.0 (StatSoft Inc, Tulsa, OK, USA).

## Results

### Immunolocalization of LTP1 epitopes at the light microscope level

**Fig. 1:**
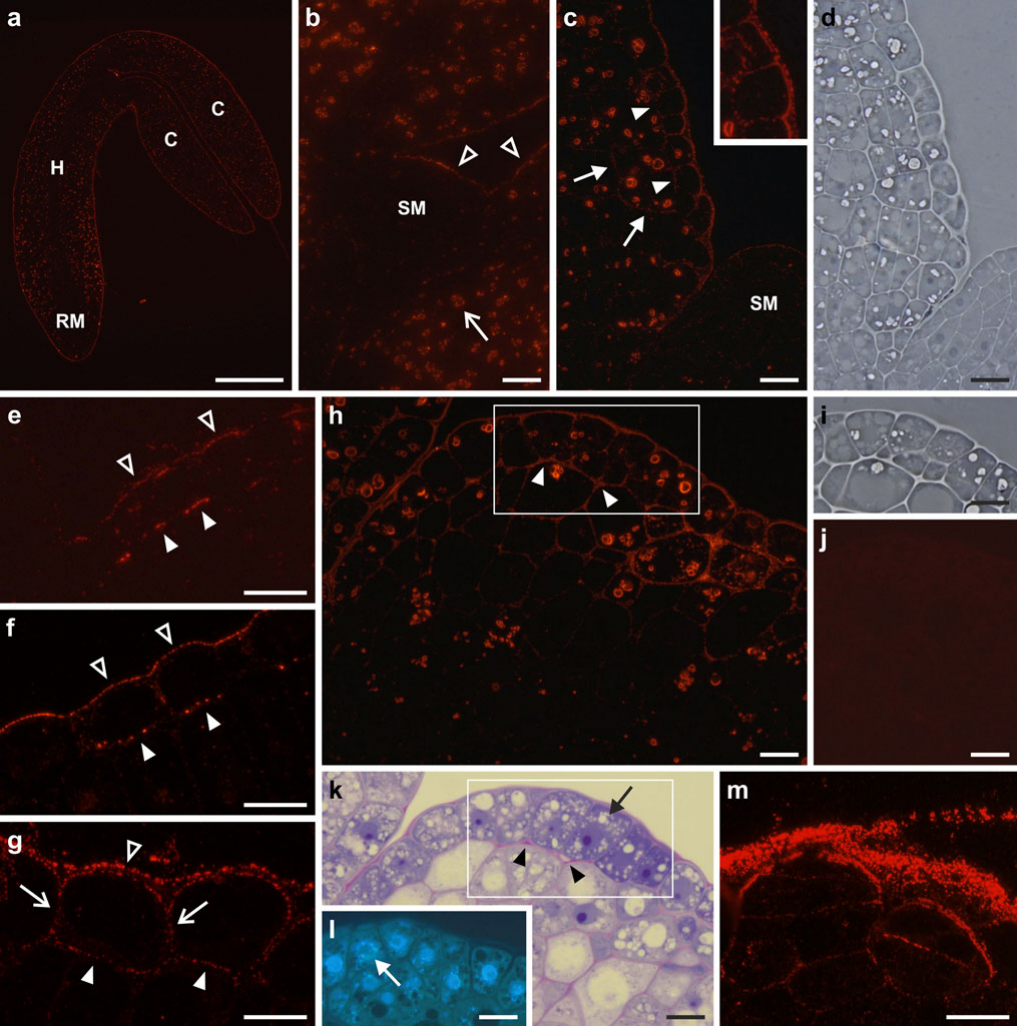
Immunofluorescence localization of LTP1 epitopes in embryogenic regions of explants at different time points of the culture. **a** Immature zygotic embryo at the start of culture, the most intense fluorescence visible on the embryo surface in all regions. **b** 1-day cultured explant, the strongest signal in the outer periclinal walls of the protoderm (*empty arrowheads*) and in plastids (*arrow*), no signal in the shoot apex. **c** 3-day cultured explant, the LTP1 epitopes most abundant in cell walls of the protoderm (*solid arrowheads*) and in external walls of subprotodermal cellular complexes (*arrows*), the *inset* shows the protoderm at higher magnification, note the labelling of the outer periclinal cell wall. **d** Phase contrast view of the section shown in **c**, clearly visible cell borders. **e**-**g** Adaxial protodermal cells of cotyledons showing intense labelling in anticlinal (*arrows*), outer periclinal (*empty arrowheads*) and inner periclinal (*solid arrowheads*) walls, day 6 (**e**), day 7 (**f**) and day 21 (**g**) of culture. **h** Proximal part of the cotyledon, day 11 of culture, labelling in the cell walls of the protoderm and subprotoderm and in the intercellular spaces (*arrowheads*). **i** Phase contrast view of the outlined area in **h**, note periclinally divided cells in the protoderm. **j** Negative control for **h**, no Cy3 labelling. **k**, **l** Sections neighbouring to the one in **h**, stained with Toluidine Blue O (**k**) and DAPI (**l**), dense cytoplasm, large nuclei and small vacuoles visible (*arrows*), *arrowheads* in **k** point to intercellular spaces between protodermal and subprotodermal cells, **l** corresponds to the *outlined areas* in **h** and **k**. **m** Periclinally divided protodermal cells displaying intense labelling within the walls, day 21 of culture. *C* cotyledon, *H* hypocotyl, *RM* root apical meristem, *SM* shoot apical meristem. *Scale bars* 100 μm (**a**), 10 μm (**b**–**m**)

**Fig. 2:**
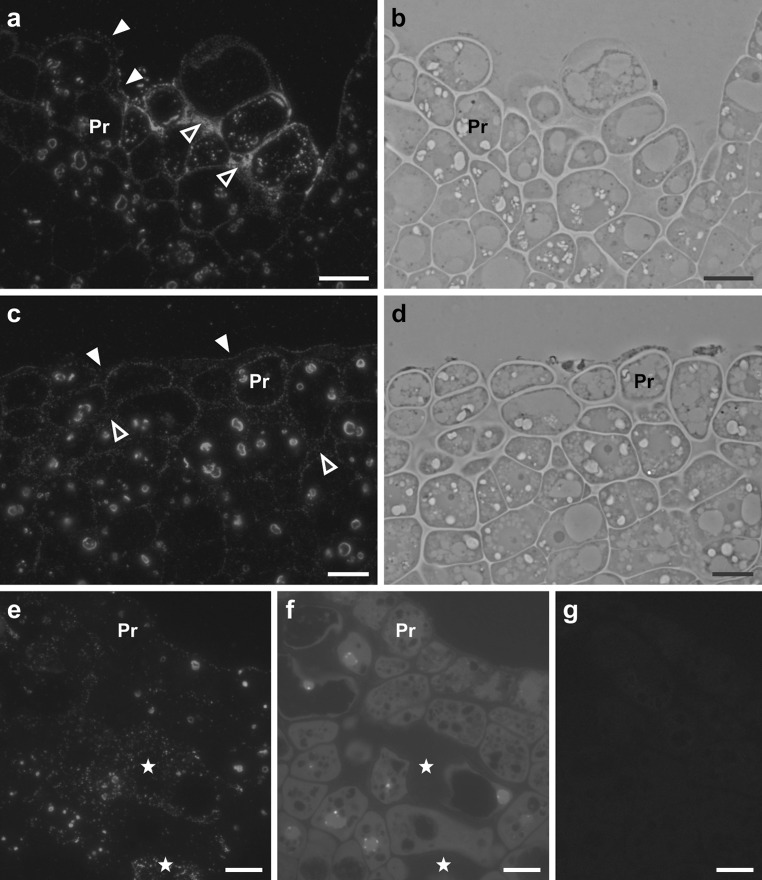
Immunofluorescence localization of LTP1 epitopes in loosened tissues of embryogenic regions. **a**, **c** Loosened protoderm of the cotyledons, labelling present on the surface of the cells (*solid arrowheads*) and in the intercellular spaces (*empty arrowheads*), day 6 (**a**) and day 7 (**c**) of culture. **b**, **d** Phase contrast views of sections shown in **a**, **c** respectively, clearly visible cell pattern. **e** Deposition of LTP1 epitopes (*asterisks*) in the spaces between loosened ground tissue cells of the cotyledon, day 11 of culture. **f** The section shown in **e** with nuclei stained with DAPI (*blue*), *asterisks* point to intercellular spaces. **g** Negative control for **e**, no labelling. *Pr* protoderm. *Scale bars* 10 μm (**a**–**g**)

In all explants, independent of the day of culture, LTP1 epitopes were present in the outer periclinal walls of protodermal cells (Fig. 1a–c, e–h). However, both the character and intensity of labelling changed with time. Immature zygotic embryos (Fig. 1a) as well as 1-day (Fig. 1b) and 2-day (not shown) cultured explants displayed LTP1 labelling mainly within the outer periclinal walls of protodermal cells. In some explants, at the start of the culture, a weak signal of a punctate character was observed to occur in anticlinal and inner periclinal cell walls of the protoderm, as well as in the cell walls of ground tissue (not shown). Swelling at the proximal end of the cotyledons (third day of culture onwards) appeared to be associated with an increase in signal intensity in the outer periclinal walls of the adaxial protoderm of the region, and with the occurrence of clear labelling within both anticlinal and inner periclinal walls (Fig. 1c, arrowheads). By this stage of development, the character of the signal in the outer periclinal walls of the protoderm had bifurcated into two distinct lines of label, facing the outer and inner regions of the wall, respectively (Fig. 1c, inset). From day 3 of culture onwards (in most cases), a strong signal was also detected in the external walls of subprotodermal cell complexes (Fig. 1c, arrows). Observation by the phase contrast microscopy revealed some characteristic cytological features of both protodermal and subprotodermal cells, namely central enlarged nuclei, small vacuoles and thickened cell walls (Fig. 1d).

On successive days of culture, the labelling of protodermal cell walls in the embryogenic region of the explant became progressively more distinct (Fig. 1e–h). The signal was present both in single cells (Fig. 1e–g), or throughout the protoderm (Fig. 1h). Its character was either punctate, both in anticlinal and inner periclinal walls (Fig. 1e–g) or continuous, mostly in inner periclinal walls (Fig. 1h). Labelled cells contained a large nucleus with a prominent nucleolus, dense cytoplasm and highly fragmented vacuome as shown in sections through an 11-day explant (Fig. 1k, l, arrows). Signal was also present in walls of a subsection of subprotodermal cells localized in the swollen proximal part of the cotyledons (Fig. 1h, day 11 of culture). Local intercellular spaces formed between layers of protodermal and subprotodermal cells also exhibited strong signal (Fig. 1h, arrowheads). Toluidine Blue O staining resulted in pink colouring in this region, indicating the possible presence of pectins (Fig. 1k, arrowheads). Protodermal cells within embryogenic regions of explants at different time points (for details see caption to Fig. 1) of the culture underwent asymmetrical periclinal divisions and such atypical cellular events were in some cases associated with an increase in signal intensity within the newly inserted walls (Fig. 1h, m).

As the explants increased in volume, a specific loosening of the peripheral cell layers was observed (Fig. 2). The continuity of the adaxial protoderm of the swollen embryogenic region was broken (Fig. 2a–d) and in walls of the loosened cells an intense signal (continuous or punctate) was observed to be present (Fig. 2a, c). Signal also occurred outside the cells: either on the surface of the protodermal cells (weaker punctate fluorescence, Fig. 2a, c, solid arrowheads) or in the intercellular spaces between cells (strong solid signal, Fig. 2a, as well as weak punctate signal, Fig. 2c, empty arrowheads). In phase contrast micrographs (Fig. 2b, d) of the sections shown in Fig. 2a, c the spaces between the loosened protodermal cells are clearly visible. In older explants (second and third week of culture), a distinct loosening was also observed in ground tissue layers of the cotyledons (Fig. 2e, f). This was accompanied by the formation of large intercellular spaces in which the signal generated by the anti-AtLTP1 antibody was very intense (Fig. 2e, asterisks). Both DAPI staining (Fig. 2f) and phase contrast microscopy (not shown) revealed the absence of nuclei within the labelled areas, indicating the extracellular deposition of the protein.

**Fig. 3:**
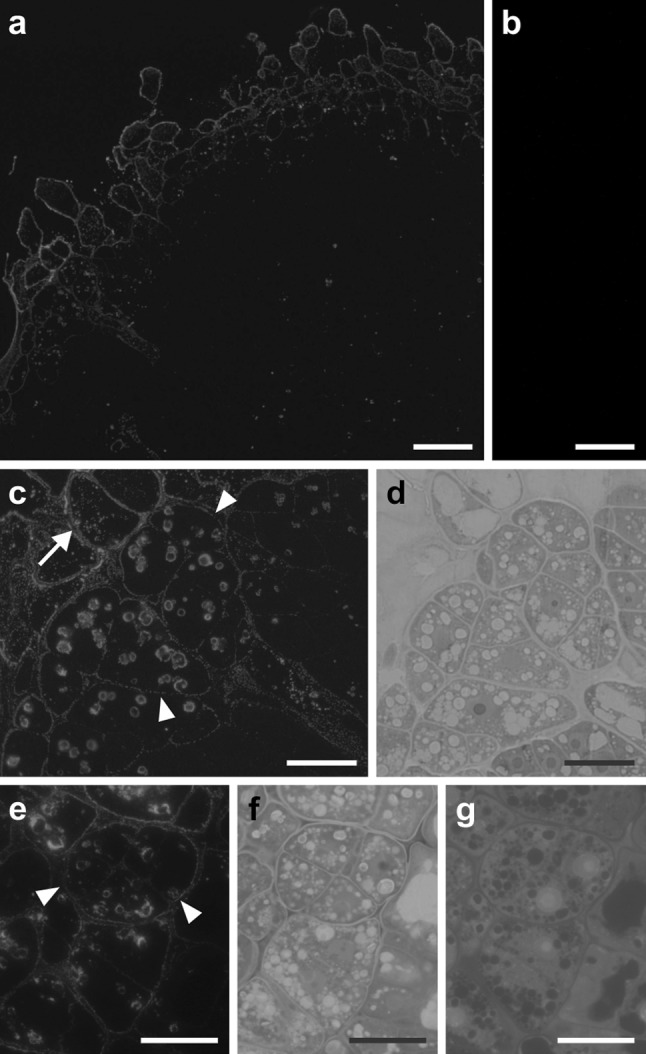
Immunofluorescence localization of LTP1 epitopes in callus and embryogenic cell complexes. **a** A section of the cotyledon of an explant cultured for 15 days, label restricted to the callus-coated periphery of the cotyledon. **b** Negative control for **a**, no labelling visible. **c**, **e** Detailed views of the cotyledon periphery, LTP1 epitopes present in the thickened cell walls enclosing embryogenic-like complexes (*arrowheads*), the recently formed walls are less intensely labelled, strong signal in the walls and cytoplasm of callus cells (**c**, *arrow*). **d**, **f**, **g** Consecutive sections of samples shown in **c**, **e**, stained with Toluidine Blue O (**d**, **f**) and DAPI (**g**), showing cytological characteristics of cells within complexes. *Scale bars* 50 μm (**a**, **b**), 20 μm (**c**–**g**)

Significant labelling was associated with callus formed by explants cultured for 10–15 days (Fig. 3a). In the callus cells, the signal was localized both in the walls and cytoplasm (Fig. 3a, c). LTP1 epitopes were also secreted outside the cells into the medium (Fig. 3c). On the surface of the callused cotyledons, there were characteristic groups of cells of common origin (Fig. 3c–g) clearly distinguishable by their thickened external walls. The walls of all cells in these embryogenic complexes exhibited a punctate labelling pattern. Moreover, the intensity of this labelling correlated with the thickness of the walls and it was strongest in walls delimiting the complexes (Fig. 3c, e). In the micrographs of sections stained with either Toluidine Blue O (Fig. 3d, f) or DAPI (Fig. 3g) cut in series with those presented in Fig. 3c, e, a large nucleus with a prominent nucleolus, small vacuoles and numerous amyloplasts are all clearly visible.

Explant cell walls at the shoot apex (Fig. 1b, c) and other non-embryogenic regions, such as hypocotyls and the distal parts of cotyledons generally displayed weak labelling. In the majority of cells, independent of the localization within the explant, strong fluorescence associated with plastids was clearly visible (Figs. 1b, c, h, 2a, c, 3c). Control sections treated without the primary antibody displayed no signal (examples presented in Figs. 1j, 2g, 3b).

**Fig. 4:**
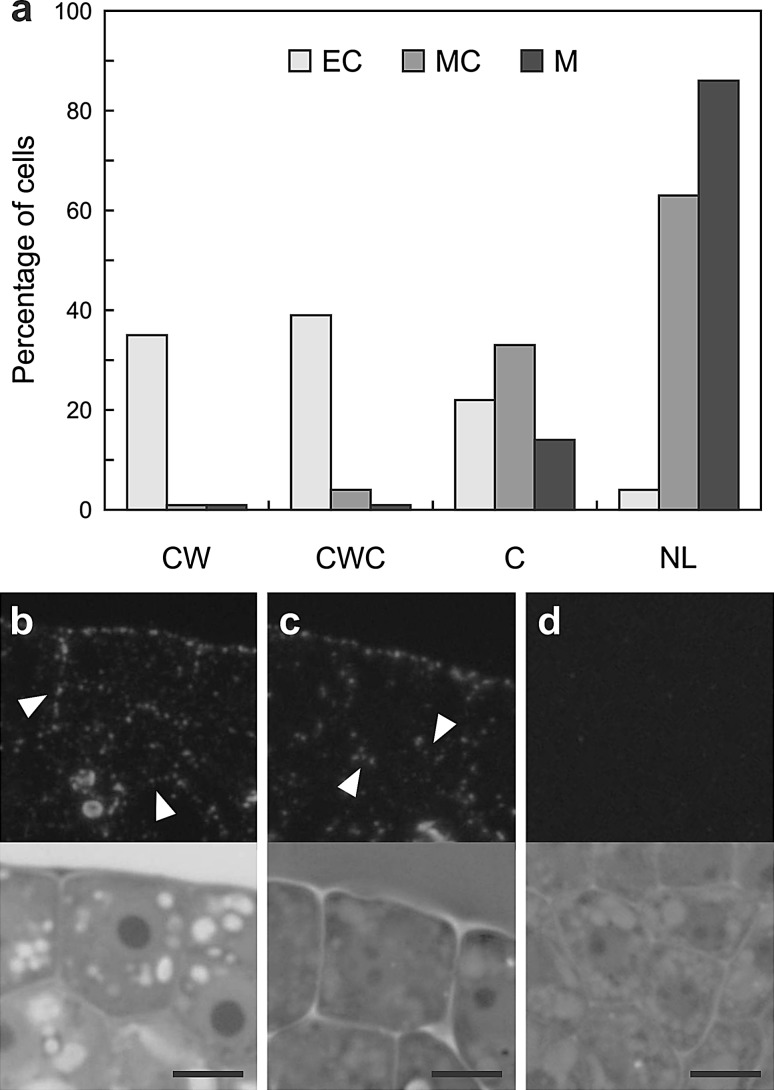
The signal localization in embryogenic and meristematic cells of the explants. **a** Histogram illustrating frequencies of the signal presence in specific localizations of various types of cells. **b** Protodermal cell of the cotyledon exhibiting features typical of embryogenic cells (*EC*), signal in the anticlinal and inner periclinal walls (*arrowheads*) and in the cytoplasm. **c** Protodermal cell of the cotyledon exhibiting features typical of meristematic cells (*MC*), scattered labelling in the cytoplasm (*arrowheads*). **d** Meristematic cells of the shoot apical meristem (*M*), no labelling. Upper micrographs in **b**, **c**, **d** show immunofluorescence images and lower micrographs show corresponding bright field and phase contrast images. *CW* cell wall labelling, *CWC* cell wall and cytoplasm labelling, *C* cytoplasm labelling, *NL* no labelling. The number of cells per group was as follows: *EC* 23, *MC* 24, *M* 22. *Scale bars* 5 μm (**b**–**d**)

Figure 4 shows the results of signal localization analysis in both embryogenic and meristematic cells of explants. Cells containing one large nucleolus, which is a feature typical of embryogenic cells (Verdeil et al. 2007), exhibited strong fluorescence in both cell walls and the cytoplasm (Fig. 4a, b). In the majority of meristematic cells with nuclei containing more than one nucleolus (Verdeil et al. 2007), found both in embryogenic regions of cotyledons and in the shoot apical meristem, no signal was involved (Fig. 4a, d). However, there were several cases in which the signal was observed only in cytoplasm (Fig. 4a, c). In a minority of meristematic cells, the signal occurred both in the cell wall and the cytoplasm (Fig. 4a). A Chi-square test of independence indicated a highly significant dependence (*p* = 0.000001) between cell type and localization of the signal within the cell.

### Co-localization of LTP1 epitopes and lipid substances within the cell walls

**Fig. 5:**
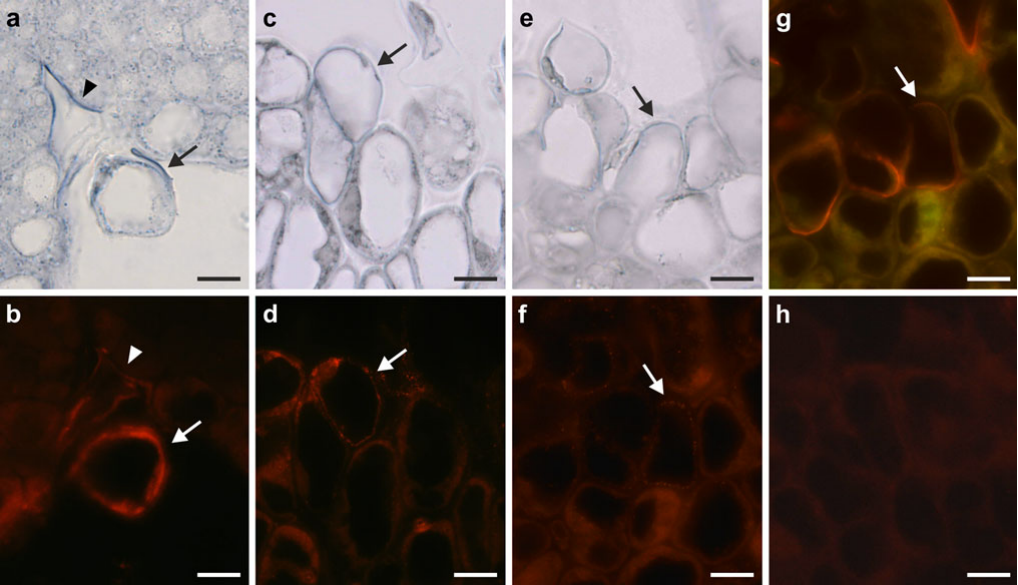
Co-localization of the lipid lamellae and the LTP1 epitopes in cells of 21-day cultured explants. *Blue colouring* (**a**, **c**, **e**) and *yellow-gold fluorescence* (**g**) of cell walls correspond to lipids stained with Sudan Black B and Nile Red, respectively, *red fluorescence* (**b**, **d**, **f**) corresponds to anti-AtLTP1 labelling. *Faint green signal* in **g** represents the autofluorescence of the tissue. **a**, **b** Detached callus cell (*arrow*), *arrowhead* points to the outer periclinal wall of the explant protoderm. **c**–**g** Surface cells of the cotyledons (*arrows*), note the loosened character of the tissue in **c**–**d**. **h** Negative control, no Cy3 fluorescence visible. *Scale bars* 10 μm (**a**–**h**)

Figure 5 shows sections through 3-week-old explants stained with Sudan Black B (Fig. 5a, c, e) which can be directly compared to consecutive sections labelled with the anti-AtLTP1 antibody (Fig. 5b, d, f). Blue colouring demonstrating the presence of lipids was observed in callus cell walls (Fig. 5a) and in walls of single cells located at the explant periphery (Fig. 5c, e). Lipid lamellae were also present at the surface of the protodermal cells (Fig. 5a) indicating that the lipid substances detected within the explant cells were chemically similar to the cuticle. After staining with Nile Red, the Sudan-positive cells exhibited yellow-gold fluorescence in their cell walls (compare Fig. 5e with g). The lipid staining was performed in conjunction with the immunocytochemistry, which revealed the presence of LTP1 epitopes in walls of the stained cells (Fig. 5b, d, f). In both callus and surface cells, signal generated by the anti-AtLTP1 antibody was detected in an outline formed by thick continuous line (Fig. 5b) or separate dots (Fig. 5d, f). Sections with the primary antibody omitted (negative control) displayed red background fluorescence easily distinguishable from the Cy3 signal (Fig. 5h).

### Immunolocalization of LTP1 epitopes at the transmission electron microscope level

**Fig. 6:**
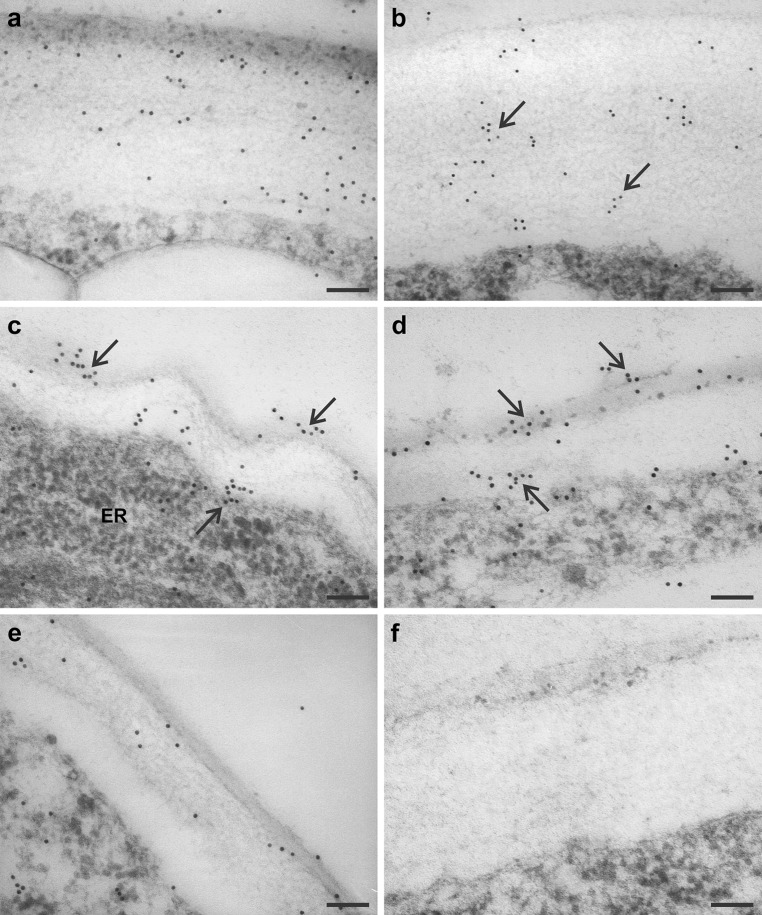
Immunogold localization of LTP1 epitopes in the outer periclinal cell walls of protoderm from embryogenic (**a**–**d**) and non-embryogenic (**e**) regions of the explants. **a**, **b** The adaxial side of the proximal region of the cotyledons, day 6 of culture, labelling present throughout the wall, distributed uniformly (**a**) or in clusters (**b**, *arrows*). **c**, **d** The adaxial side of the proximal region of the cotyledons, day 7 (**c**) and day 9 (**d**) of culture, the labelling visible in both the outer and inner part of the cell wall (*arrows*), notice the undulated cell wall in **c**. **e** Shoot apex of an explant cultured for 6 days, weak label restricted to the outer part of the cell wall. **f** Negative control, no gold particles visible. *ER* endoplasmic reticulum. *Scale bars* 100 nm (**a**–**f**)

**Fig. 7:**
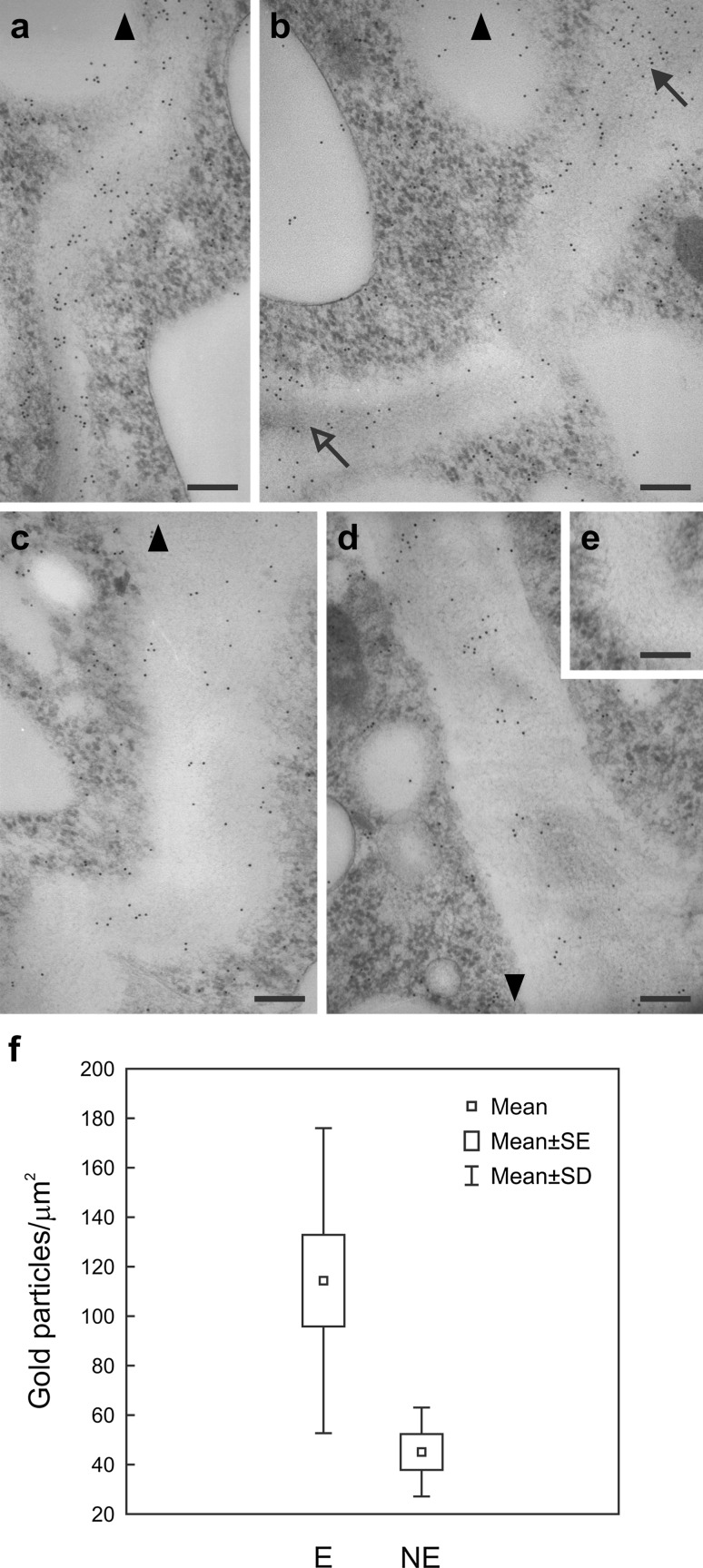
Immunogold localization of LTP1 epitopes in the anticlinal cell walls of protoderm from embryogenic (**a**, **b**) and non-embryogenic (**c**, **d**) regions of explants cultured for 6 days. **a**, **b** The adaxial side of the proximal part of the cotyledon, showing the intense gold labelling and a gradient of labelling intensity growing towards cell surface, *solid* and *open arrows* in **b** indicate the apical and basal regions of the wall, respectively. **c** The adaxial side of the distal part of the cotyledon with few gold particles. **d** The abaxial side of the cotyledon, a few gold particles gathered in small clusters in the central part of the cell wall can be observed. **e** Negative control showing no labelling. **f** Quantification of immunogold label in walls of embryogenic (*E*) and non-embryogenic (*NE*) regions of the explants, data are means (±standard error and standard deviation) of ten micrographs per each region. *Arrowheads* (**a**–**d**) point to the cell surface. *Scale bars* 200 nm (**a**–**e**)

Localization of the LTP1 epitopes in the explant tissues was examined in more detail using immunogold labelling at the transmission electron microscope level. The TEM data demonstrated that the distribution of the LTP1 epitopes within the cell walls of embryogenic and non-embryogenic regions of the explants varied in terms of both spatial pattern and abundance. These differences were most clearly observed in both periclinal and anticlinal walls of protodermal cells (Figs. 6, 7, respectively).

For example, in the adaxial protodermal cells of the proximal part of the cotyledons (embryogenic region, Fig. 6a–d) the labelling of outer periclinal walls was relatively strong, while in the protodermal cells of the shoot apex (non-embryogenic region) it was rather weak (Fig. 6e). In the cotyledons, labelling was distributed throughout the wall, either uniformly (Fig. 6a) or in small clusters (Fig. 6b). In some external cell walls, the labelling was restricted to the outer and inner regions of the wall (Fig. 6c, d). In the shoot apex, the LTP1 epitopes were detected in the outer region of the cell wall and in the cuticle (Fig. 6e).

Marked differences in the distribution of gold label were also observed between anticlinal walls of protodermal cells in the embryogenic and non-embryogenic regions of the explants (Fig. 7). The anticlinal protodermal cell walls localized on the adaxial side of the proximal part of cotyledons, accumulated more gold particles (Fig. 7a, b), whilst anticlinal walls on the adaxial side of the distal part of cotyledons (Fig. 7c), as well as those located on the abaxial side of cotyledons displayed considerably less label (Fig. 7d). It is also worth noting that in the anticlinal walls of some cells localized in the embryogenic regions of the explants (adaxial protoderm of the proximal part of cotyledons), a gradient of LTP1 epitope intensity could be observed (Fig. 7b). The labelling was strongest in regions of the wall adjacent to the explant surface, where it was localized in the so-called cuticular pegs and was weaker in the parts of walls adjacent to the subprotodermal cells (Fig. 7b). In the embryogenic region, the mean number of gold particles per μm^2^ of the wall was about 2.5-fold higher compared to the non-embryogenic region (114.35 ± 61.65 and 45.09 ± 17.98, respectively; Fig. 7f). The Student’s *t* test comparing the means showed significant differences (*p* = 0.018) between the regions.

**Fig. 8:**
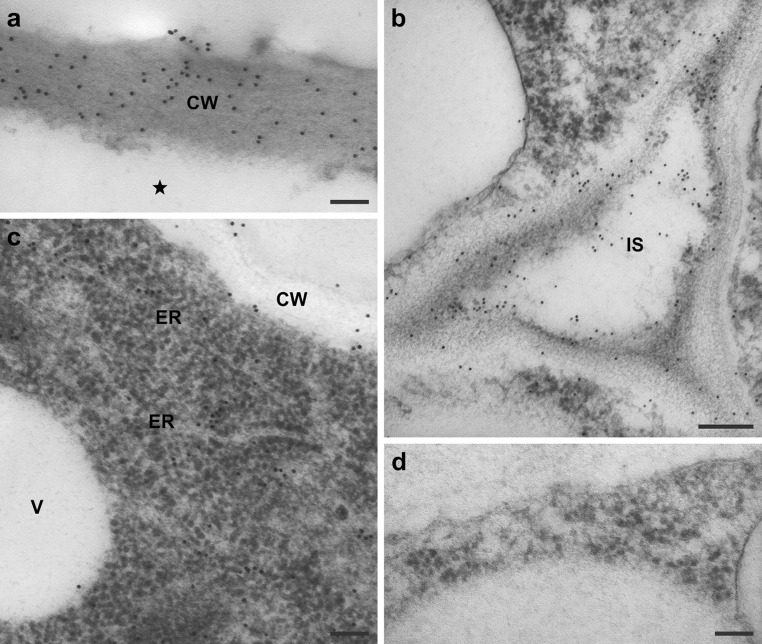
Immunogold localization of LTP1 epitopes in the cell walls and cytoplasm of the explants. **a** Callus cell from an explant cultured for 21 days, notice densely and uniformly labelled cell wall, the *asterisk* marks the cell interior. **b** Tricellular junction with intercellular space formed between the adaxial protodermal and subprotodermal cells of the cotyledon, day 4 of culture. **c** Intracellular localization of LTP1 epitopes, adaxial protodermal cell of the cotyledon, day 7 of culture. **d** Negative control, no labelling visible. *CW* cell wall, *ER* endoplasmic reticulum, *IS* intercellular space, *V* vacuole. *Scale bars* 100 nm (**a**, **c**, **d**), 200 nm (**b**)

LTP1 epitopes were present in callus cell walls, in which the labelling was uniformly distributed throughout the wall (Fig. 8a). The labelling was also observed on the edges of cell walls lining the intercellular spaces as well as within the spaces (Fig. 8b). Apart from the cell wall localization of labelling, analysis of protodermal explant cells at the ultrastructural level revealed the presence of LTP1 epitopes within the cytoplasm, in close proximity to membranes of the endoplasmic reticulum (ER, Figs. 6c, 8c), as well as within secretory vesicles located close to the cell wall and in the vicinity of formed intercellular spaces (not shown). Moreover, the LTP1 epitopes were rarely present in the vacuoles (Fig. 8c) and plastids (not shown). No accumulation of the gold particles was seen in control sections incubated with the blocking buffer instead of the primary antibody (Figs. 6f, 7e, 8d).

## Discussion

In spite of numerous and multidisciplinary research in the field, the precise biological function of lipid transfer proteins in plant cells is still unclear. Their previously suggested role in intracellular lipid trafficking (Arondel and Kader 1990) has not been confirmed in in vivo experiments, although intracellular occurrence of these proteins has been reported (Sossountzov et al. 1991; Vega-Maray et al. 2004). Functions currently proposed (for review see Yeats and Rose 2008) relate mainly to their apoplastic localization and secretion. The results presented in this paper indicate a potential involvement of LTPs in the processes of totipotency and plasticity of plant cells and thus represent a useful contribution to our understanding of this ‘puzzling family of plant proteins’ (Kader 1996).

Immunofluorescence analysis of LTP1 epitopes distribution in cells of *A. thaliana* explants (Figs. 1, 2, 3, 4) showed that these proteins are present both in the cell wall and the cytoplasm. Our TEM data (Figs. 6, 7, 8) revealed more precisely the intracellular localization of LTPs close to the ER, and inside vesicles as well as vacuoles. The presence of LTP epitopes in the vicinity of the ER is consistent with the fact that an LTP from maize is synthesized on the membrane-bound polysomes (Vergnolle et al. 1988) while detection of a signal inside vesicles localized in the vicinity of the cell wall suggests that LTPs are transported outside the cell in a vesicular manner. The observed, intense immunolabelling of the plastids is also of note. However, further studies are required to determine a precise suborganellar location of these proteins, as well as their functional role. To date, neither the LTP-mediated transfer of lipids between the ER and plastids nor between the plastid envelope and thylakoids has been confirmed in vivo (for review see Moreau et al. 1998; Benning 2008). Intracellular occurrence of LTPs has been reported previously, in a variety of tissues and species, including coleoptiles and leaves of maize (Sossountzov et al. 1991) and pollen grains of *Parietaria judaica* (Vega-Maray et al. 2004). Recent studies in *Allium* have shown that AlLTPs with both C-terminal and N-terminal signal peptides were predicted to be localized to the vacuole (Yi et al. 2009). However, our results provide direct evidence for the presence of lipid transfer proteins within dedifferentiating and redifferentiating cells, which may indicate the occurrence of membrane biogenesis during cell differentiation. It is worth noting that intense cytoplasmic signal was observed in the adaxial cells of cotyledons which exhibited either embryogenic or meristematic features (Fig. 4), i.e. in the regions of explant where a change of direction of the cell differentiation was taking place.

Extracellular localization of the LTPs, mainly in the cell wall, has been reported in previous studies (Carvalho et al. 2004; Thoma et al. 1993; Tsuboi et al. 1992). In *A. thaliana*, their presence was observed in walls of cells from various regions of the plant body, for example in the epidermis and cortex of leaves, petioles and stems (Thoma et al. 1993). Studies of *LTP* gene expression in carrot (Sterk et al. 1991) and *Arabidopsis* (Vroemen et al. 1996) demonstrated that *LTP* transcripts are mainly localized in protoderm. In carrot, *LTP* expression was identified as a molecular marker for protoderm formation (Sterk et al. 1991), and in *Arabidopsis* the *AtLTP1* gene was expressed in protoderm in the early globular stages of embryo development (Vroemen et al. 1996). Our studies are concerned with similar stages of the plant development. However, they are focused on the distribution of protein epitopes, not gene expression. These data demonstrate for the first time the spatio-temporal distribution of LTP1 protein epitopes in whole explants, during somatic embryogenesis in *A. thaliana*.

Analysis of the signal localization in protodermal and subprotodermal cells of proximal regions of cotyledons demonstrated that the majority (74 %) of cells exhibiting features typical of embryogenic cells were labelled, either in the cell wall or both in wall and the cytoplasm (Fig. 4a, b). Meristematic-like cells of the cotyledons showed no labelling or exclusively cytoplasmic labelling (Fig. 4a, c). Canhoto et al. (1996) reported that in pineapple guava it was impossible to find any particular characteristics of the meristematic-like cells formed in cotyledons before they had become embryogenic that would distinguish them from meristematic-like cells not involved in the embryogenic process. According to our results (Fig. 4), during somatic embryogenesis in *Arabidopsis* LTP epitopes detected in cotyledonary meristematic cells may indicate that these cells have entered the embryogenic pathway, but this hypothesis requires additional studies.

Our observation that LTP1 epitopes are abundant in the walls of callus cells and in cells which increase in volume, or change their shape (Figs. 2a, 3a, 8a) is of interest in relation to a putative role/involvement for LTPs in wall loosening (Nieuwland et al. 2005). The results obtained by Nieuwland et al. (2005) showed that the availability of LTPs to engage in hydrophobic interactions would be essential for a possible role in cell wall loosening. It is postulated that after secretion, the hydrophobic cavity of LTPs may interact with hydrophobic molecules in the cellulose/xyloglucan network and that this complex, with its charged surface, could disrupt the surrounding hydrogen bonds between the cellulose and hemicellulose (Nieuwland et al. 2005). Our data are also in accordance with a previous observation (Sabala et al. 2000) which showed that cells exhibiting overexpression of LTPs can elongate, whereas cell clusters with underexpression of LTPs cannot. It was also demonstrated by Mollet et al. (2000) that LTPs may interact with other wall components. Their research revealed that in lilies, a lipid transfer-like protein in conjunction with pectic polysaccharide is necessary for proper pollen tube adhesion on the stylar matrix. Our observations seem to support this hypothesis: as shown in Fig. 1h and k; in that the intercellular spaces formed between layers of protodermal and subprotodermal cells contained an abundance of both LTP epitopes and pectins.

With regard to LTP epitopes distribution within the explant cell walls during somatic embryogenesis, we wish to highlight two observations. Firstly, if we take into consideration that LTPs participate in the delivery of lipid substances into the cuticle, we can assume that the lipid substance deposition observed in the walls of cells possibly undergoing somatic embryogenesis is ‘atypical’ for protodermal cells. In ‘normal’ conditions, a cuticle is present only in the outer periclinal walls. This means that the protodermal explant cells with LTP epitopes present in both inner and outer periclinal, as well as anticlinal walls (Fig. 1c, e–h), may have changed their phenotype. Of course, this does not prove that such cells are proembryogenic, but this is in accordance with our previous observation (Kurczyńska et al. 2007) that the protodermal cells (in the culture system used) participate in somatic embryo formation. In the course of embryogenesis, the cells undergo atypical (Considine and Knox 1981) periclinal divisions (Fig. 1h and m, see also: Kurczyńska et al. 2007) which means that their phenotype changes. As shown in Fig. 1h and m, either of the two cells formed as a result of periclinal division exhibited labelling in their cell walls. However, it is still unclear as to whether or not this has an influence on the future fate of these cells. Very high levels of the LTP epitopes were also observed in some callus cells (Figs. 3a, 8a) and other cell phenotypes present in the explants. This also may suggest that the cells in which the LTP epitopes are present change their direction of differentiation.

The second important observation is that the presence of the lipid lamellae within some cell walls is correlated with high levels of LTP epitopes in these cell walls (Fig. 5). Such lipid substances can be considered as a cutin deposited within the wall. Whether or not such cells exhibit a protodermal/epidermal phenotype or a periderm phenotype is unknown; further studies on the ultrastructural level are required to answer the question as to whether the lipid lamella is deposited outside the cell wall or inside it. Nevertheless, a previous study on *H. lupulus* has shown that cells or group of cells committed to express morphogenic competence are surrounded by cutin (Fortes et al. 2002). The authors postulated that this is necessary to create a specific environment with an altered permeability and receptor content. The data presented here also indicate that the cells which change their fate may require separation from their neighbours to stop the flow of ‘information’ from surrounding cells. In studies of *Camellia* leaves, the presence of cutin in the wall was postulated to be a necessary factor for entering the somatic embryogenesis pathway (Pedroso and Pais 1995).

Individual cells of explant cotyledons enclosed in cell walls enriched in LTPs and/or lipid substances and thus physiologically isolated, act as meristemoids differentiating into somatic embryos. They are densely cytoplasmic and are able to undergo asymmetric cell divisions. In the present study, the isolation of the meristemoids by cell wall modifications occurred early in their development, before the formation of a partition wall (Fig. 1e–g). Thickened modified walls were also observed around the multicellular cell complexes arising after a few rounds of meristemoid division (Fig. 3c, e). Similarly, during the early development of the *Origanum dictamnus* glandular scales, the progressive cutinization of the stalk cell lateral walls takes place (Bosabalidis and Tsekos 1982). The salt glands of *Frankenia pauciflora* are encapsulated in a cuticular envelope consisting of Sudan-positive lipids (Olesen 1979) and in *Heliamphora folliculata*, the nectary constitutes an apoplastic field with cells isolated from ‘ordinary’ parenchyma cells by cutinized walls lacking plasmodesmata (Płachno et al. 2007). Moreover, several previous studies have revealed a high proportion of LTP transcripts in mature meristemoid-derived structures such as guard cells and trichomes (Aziz et al. 2005; Smart et al. 2000).

Further molecular studies are required to answer the question as to what form(s) of LTP are present during the somatic embryogenesis in *Arabidopsis*. An analysis of genes involved in acyl lipid metabolism showed that in *Arabidopsis* there are 71 putative LTPs, which are classified into eight categories based upon a conserved Cys pattern (Beisson et al. 2003). All LTPs upregulated in the growing epidermis, belong to type 1 and 5 and are postulated to be involved in the cuticle synthesis; type 3, however, may function as signalling molecules in the apoplast (Maldonado et al. 2002; Suh et al. 2005).

In summary, our data have demonstrated that the distribution of LTP epitopes changes both spatially and temporally during somatic embryogenesis in *Arabidopsis* explants. LTP epitopes were shown to be more abundant in the cell walls of the protoderm and subprotoderm located in the regions of the explants which are likely to become embryogenic. These results thereby suggest that such proteins may be involved in the morphogenic changes taking place during somatic embryogenesis. The LTP epitopes were also shown to be abundant within the walls of cells which changed their shape, volume or direction of cell divisions, which suggests that the presence of LTP epitopes is correlated with changes in cell fate. Furthermore, the presence of LTP epitopes in conjunction with lipid lamellae within the cell wall may serve as a marker for cells which will change their direction of differentiation.

## Abbreviations

ER: Endoplasmic reticulum
LTP: Lipid transfer protein
PBS: Phosphate-buffered saline
SE: Somatic embryogenesis
